# The association between history of prenatal loss and maternal psychological state in a subsequent pregnancy: an ecological momentary assessment (EMA) study

**DOI:** 10.1017/S0033291721002221

**Published:** 2023-02

**Authors:** Claudia Lazarides, Nora K. Moog, Glenn Verner, Manuel C. Voelkle, Wolfgang Henrich, Christine M. Heim, Thorsten Braun, Pathik D. Wadhwa, Claudia Buss, Sonja Entringer

**Affiliations:** 1 Institute of Medical Psychology, Charité – Universitätsmedizin Berlin, corporate member of Freie Universität Berlin, Humboldt-Universität zu Berlin, Berlin, Germany; 2 Faculty of Life Science, Department of Psychology, Psychological Research Methods, Humboldt-University of Berlin, Berlin, Germany; 3 Department of Obstetrics, Charité – Universitätsmedizin Berlin, corporate member of Freie Universität Berlin, Humboldt-Universität zu Berlin, Berlin, Germany; 4 Development, Health and Disease Research Program, University of California, Irvine, CA, USA; 5 Department of Pediatrics, University of California, Irvine, CA, USA

**Keywords:** Ecological momentary assessment, linear mixed modeling, mood, pregnancy, prenatal loss, stress

## Abstract

**Background:**

Prenatal loss which occurs in approximately 20% of pregnancies represents a well-established risk factor for anxiety and affective disorders. In the current study, we examined whether a history of prenatal loss is associated with a subsequent pregnancy with maternal psychological state using ecological momentary assessment (EMA)-based measures of pregnancy-specific distress and mood in everyday life.

**Method:**

This study was conducted in a cohort of *N* = 155 healthy pregnant women, of which *N* = 40 had a history of prenatal loss. An EMA protocol was used in early and late pregnancy to collect repeated measures of maternal stress and mood, on average eight times per day over a consecutive 4-day period. The association between a history of prenatal loss and psychological state was estimated using linear mixed models.

**Results:**

Compared to women who had not experienced a prior prenatal loss, women with a history of prenatal loss reported higher levels of pregnancy-specific distress in early as well as late pregnancy and also were more nervous and tired. Furthermore, in the comparison group pregnancy-specific distress decreased and mood improved from early to late pregnancy, whereas these changes across pregnancy were not evident in women in the prenatal loss group.

**Conclusion:**

Our findings suggest that prenatal loss in a prior pregnancy is associated with a subsequent pregnancy with significantly higher stress and impaired mood levels in everyday life across gestation. These findings have important implications for designing EMA-based ambulatory, personalized interventions to reduce stress during pregnancy in this high-risk group.

## Background

The prevalence among women of childbearing age of prenatal loss, the loss of an unborn child during pregnancy through miscarriage or stillbirth, is substantial, with one out of five women experiencing a miscarriage (loss of an embryo or fetus before the 20th week of gestation), and one out of 160 women experiencing a stillbirth (loss of a fetus occurring after the 20th week of gestation and a weight above 500 g) (Blencowe et al., 2016; El Hachem et al., 2017; Lawn et al., 2016; MacDorman, Kirmeyer, & Wilson, 2012; Murphy et al., 2017; Price, 2006). The negative consequences on women's mental health of losing an unborn child have been reported in several studies: prenatal loss is related to a higher risk for psychiatric disorders such as post-traumatic stress disorder, anxiety disorders, and major depression (reviewed in Engelhard, van den Hout, & Arntz, 2001; Farren et al., 2016, 2018, 2020; Horesh, Nukrian, & Bialik, 2018; Hughes, Turton, & Evans, 1999; Jacob, Polly, Kalder, & Kostev, 2017; Turton, Evans, & Hughes, 2009). Over 80% of women who experience prenatal loss become pregnant within the subsequent 12-month period (Regan et al., 2019; Sundermann, Hartmann, Jones, Torstenson, & Velez Edwards, 2017), and it is therefore likely that the negative effects of prenatal loss on maternal psychological well-being may extend to the subsequent pregnancy. Given the prominent role of maternal psychological state during pregnancy in many critical pregnancy, birth and offspring developmental and health outcomes (Bale et al., 2010; Buss, Entringer, & Wadhwa, 2012; Entringer, 2013; Entringer, Buss, & Wadhwa, 2012; Entringer, de Punder, Buss, & Wadhwa, 2018; Giannandrea, Cerulli, Anson, & Chaudron, 2013; Heim, Entringer, & Buss, 2019; Wadhwa, Entringer, Buss, & Lu, 2011), it is crucially important to determine the relationship of a previous prenatal loss on maternal psychological well-being during a subsequent pregnancy.

Previous studies on the association between prenatal loss and maternal psychological state in a subsequent pregnancy have focused primarily on maternal anxiety and depression (Hughes et al., 1999; Hunter, Tussis, & MacBeth, 2017; Turton et al., 2009). These previous studies have several limitations. First, the majority of these studies have focused on clinical diagnoses of psychiatric disorders (Blackmore et al., 2011; Gong et al., 2013; Turton, Hughes, Evans, & Fainman, 2001), thereby precluding the ascertainment of whether this relationship is evident with variation in maternal psychosocial distress and affective state along a continuum, potentially below clinical thresholds. The clinical relevance of maternal psychological state in pregnancy is not restricted to psychopathology but is evident along a continuum (reviewed in Burgueno, Juarez, Genaro, & Tellechea, 2020; Graignic-Philippe, Dayan, Chokron, Jacquet, and Tordjman, 2014; Lautarescu, Craig, & Glover, 2020; Tarabulsy et al. 2014; Wadhwa et al. 2011; Walsh et al. 2019).

Second, previous study on the association of prenatal loss with maternal psychological well-being has relied exclusively on the use of traditional, retrospective recall-based measures to characterize maternal psychological state (for a meta-analytic overview, refer Campbell-Jackson & Horsch, 2014; Hunter et al. 2017). Respondents are typically asked to rate how stressed, anxious, or depressed they have felt over the past week/month/since the beginning of their pregnancy. These traditional measures are prone to retrospective recall bias (Podsakoff, MacKenzie, & Podsakoff, 2012), thereby limiting their validity. In addition, most participants are asked to fill out the questionnaires in either a clinical or research laboratory setting, thereby potentially limiting their generalizability (ecological validity) to everyday real-life situations and circumstances.

Third, the majority of previous studies have incorporated only one measurement time point, in either early or late pregnancy; only 4 of 19 previous studies have used a longitudinal study design (Hunfeld, Agterberg, Wladimiroff, & Passchier, 1996; Robertson-Blackmore et al., 2013; Tsartsara & Johnson, 2006; Woods-Giscombe, Lobel, & Crandell, 2010; refer recent meta-analysis by Hunter et al., 2017). It may be particularly important to assess maternal psychological state longitudinally across pregnancy because the association between history of prenatal loss and maternal psychological state may change across the course of gestation. Once the critical hallmark of 20th week of gestation is passed the risk for prenatal loss decreases significantly (ACOG, 2018; Ammon Avalos, Galindo, & Li, 2012; Mukherjee, Velez Edwards, Baird, Savitz, & Hartmann, 2013), potentially contributing to improvements in maternal well-being in the second half of pregnancy. This issue may have clinical relevance because studies of the effects of maternal stress during pregnancy have reported differential effects depending on the gestational time window of its occurrence (Buss et al., 2009, 2012a, 2012b; Davis, Head, Buss, & Sandman, 2017; Entringer et al., 2016).

Fourth, several of the previous studies are limited in terms of study design, particularly the lack of appropriate comparison groups. For example, some studies have included in the comparison group a combination of women who were pregnant for the first time and also women who were pregnant at least one time before the current (index) pregnancy, whereas the group of women with a history of prenatal loss include, obviously, only multigravida (women who were pregnant at least once before), and these studies did not adjust for gravida or parity status (Abbaspoor, Razmju, & Hekmat, 2016; Bicking Kinsey, Baptiste-Roberts, Zhu, & Kjerulff, 2015; Cumming et al., 2007; Farren et al., 2016, 2018; Volgsten, Jansson, Svanberg, Darj, & Stavreus-Evers, 2018). Because the event/experience of a prior pregnancy might be associated with biological and psychological changes (Armstrong, Hutti, & Myers, 2009), this could potentially confound the association between history of prenatal loss and psychological state in a subsequent pregnancy. We note that in the current study we addressed this issue by including parity status as a covariate in all analysis. In addition, we conducted a sensitivity analysis by examining the effect of prenatal loss on our study outcomes in the study's subpopulation of multigravid women.

Fifth, several studies have failed to account for other important potential confounders associated with both risk for prenatal loss and impairments in mental well-being, such as sociodemographic factors (e.g. income, maternal age) and obstetric characteristics (e.g. obstetric risk factors; Blackmore et al., 2011) EMA methods can address several of these above-discussed limitations by employing repeated real-time measurements of psychological states in participants' natural daily environments, thereby minimize biases associated with retrospective recall measures to provide more accurate and ecologically valid measures of psychological/behavioral states (Smyth & Stone, 2003). Thus, the aim of the current study was to examine the association of history of prenatal loss with assessments during a subsequent pregnancy in early as well as late gestation of maternal psychological state (maternal momentary pregnancy-specific distress and mood) using EMA methods.

## Materials and methods

### Participants

The study was conducted at the Institute of Medical Psychology and the Department of Obstetrics at the Charité Universtitaetsmedizin Berlin, Germany. Women with a singleton, intrauterine pregnancy were recruited prior to 16 weeks gestation. Exclusion criteria were twin pregnancies, uterine, placental/cord anomalies, fetal congenital malformations, and systemic corticosteroid intake. The study protocol included two study visits at the laboratory during early (*T*1: 12–16 weeks gestation) and late pregnancy (*T*2: 30–34 weeks gestation), followed by a 4-day EMA period, as described below. The Charité Institutional Review Board approved the study, and all participants provided written, informed consent.

The characteristics of the study participants are presented in Table 1. Miscarriage was defined as the loss of an unborn child during a recognized pregnancy before the 20th week of gestation, and if gestational age (GA) is not available, fetal weight equal or below 500 g (Farquharson, Jauniaux, Exalto, & Pregnancy, 2005). Prenatal losses occurring after the 20th week of gestation and a weight above 500 g were termed stillbirths (Tavares Da Silva et al., 2016). In total, 25.8% of the participating women reported the experience of prenatal loss, either miscarriage or stillbirth, in a previous pregnancy (*N* *=* 40). 5.8% reported two prenatal losses, and 1.9% reported more than two prenatal losses (*N* = 3) in their reproductive history. The prevalence of obstetric complications during pregnancy was low in our study population (5.8%).

**Table 1.** Maternal sociodemographic and obstetric characteristics

Maternal characteristics	*N* = 155
GA at assessment (weeks gestation, *M* *±* s.d.)	
T1 – early gestation	14.4 ± 1.5
T2 – late gestation	32.5 ± 1.3
Maternal age (years, *M* *±* s.d.)	32.5 ± 4.7
Highest degree (*n*, %)	
No degree	14 (9.0)
Technical or vocational training	44 (28.3)
Bachelor degree	23 (14.8)
Master degree	54 (34.8)
Ph.D.	20 (12.9)
Total monthly net household income [*M* *±* s.d., *in Euro, categories below*^a^ *n* (%)]	3994 *±* 2411
<1250	11 (7.1)
1250–1749	6 (3.9)
1750–2249	10 (6.5)
2250–2999	23 (14.9)
3000–3999	37 (24.0)
4000–4999	26 (16.9)
>5000	41 (26.6)
Country of birth (*n*, %)	
Germany	127 (82.5)
Obstetric characteristics	
History of prenatal loss (*n*, %) – yes	40 (25.8)
0	115 (74.2)
1	28 (18.1)
2	9 (5.8)
>2	3 (1.9)
Parity	
Nulliparous	95 (61.2)
Multiparous	60 (38.7)
Gravidity	
1	76 (49.0)
2	44 (28.4)
3	17 (11.0)
⩾4	18 (11.6)
Obstetric risk factors (*n*, %)	9 (5.8)
Gestational diabetes	4 (2.6)
Hypertension	2 (1.3)
Preterm labor	4 (2.6)

*Note:* Due to rounding, some totals may not correspond with the sum of the separate figures.

a income ranges based on KIGGs study's sociodemographic index (Lampert, Hoebel, & Kuntz, 2018).

### Measures

#### Maternal characteristics

At each visit, trained study personnel conducted structured interviews to obtain information on sociodemographic characteristics and reproductive history (e.g. gravidity, parity, number of previous pregnancy losses), and estimated date of conception. GA at visit was computed based on early ultrasound measurements. Data on obstetric risk factors were abstracted from the medical record.

#### EMA-based measures of maternal psychological state

We assessed momentary maternal pregnancy-specific distress and mood using an EMA protocol for ambulatory, real-time measurement of affective states. The EMA protocol was delivered through the mobile phone application *movisensXS* (movisensXS; movisens, 2020). The 4-day EMA protocol spanned two consecutive weekdays and a weekend (Thursday–Sunday, or Saturday–Tuesday). Participants were provided a smartphone with an electronic diary application. Throughout the EMA period, participants were prompted on average eight times per day (prompts were between 30 and 90 min apart between the hours of 8AM and 8PM).

Pregnancy-specific distress (PSD_mean; Rini, Dunkel-Schetter, Wadhwa, & Sandman, 1999) was assessed by inquiring about the woman's feelings (happiness and ambivalence) about being pregnant, her concerns about the baby's health, bodily discomfort due to pregnancy-related changes, and concerns about giving birth. Women rated these items on a Likert scale ranging from 0 to 5 (‘not at all’ to ‘completely’). Participants' ratings at each prompt were aggregated across all items and average scores were computed. The measure of pregnancy-specific stress was chosen for this study because measures that assess distress in a specific area of life may better reflect individual responses to these conditions than global stress questionnaires (Bussières et al., 2015; Stanton, Lobel, Sears, & DeLuca, 2002). This pregnancy-specific distress measure has previously been linked to pregnancy and offspring outcomes (e.g. Buss, Davis, Muftuler, Head, & Sandman, 2010; Glynn, Schetter, Hobel, & Sandman, 2008).

Maternal momentary mood was measured by the multidimensional mood questionnaire (MDBF) developed for daily diary research and validated for EMA studies (Courvoisier, Eid, & Lischetzke, 2012; Courvoisier, Eid, Lischetzke, & Schreiber, 2010; Hinz, Daig, Petrowski, & Brahler, 2012; Steyer, Schwenkmezger, Notz, & Eid, 1994). Participants rated their momentary mood along three dimensions: valence (good–bad mood, GB), arousal (calmness–nervousness, CN), and tiredness (alertness–tiredness, AT) on 12 items, four items for each dimension, with a balanced number of negatively worded and positively worded items. Items were rated on a 6-point Likert scale ranging from 0 to 5 (‘not at all’ to ‘completely’). Positively worded items were reversed before aggregating answers to derive an average score for each dimension (good–bad mood: GB_mean, calm–nervous: CN_mean, alert–tired: AT_mean) for each prompt. The derived original scores were reversed to ease the interpretation of the results such that higher average scores for each dimension indicate an unfavorable affective state (bad mood, nervous, tired), and lower average scores indicate favorable affective states (good mood, calm, alert).

### Statistical analysis

We performed all statistical analyses in R version 3.5.1 (R Development Core Team, 2018). The R-package nlme version 3.1-137 was used for linear mixed model analyses (Pinheiro, Bates, DebRoy, Sarkar, & R Core Team, 2018).

#### Variance decomposition of momentary measures

We used linear mixed(-effect) models (LMMs; cf. multilevel models) to identify the proportion of variance at the different levels of the data (momentary, day, stage of pregnancy and participant level; Snijders & Bosker, 2012) with regard to pregnancy-specific distress and the three mood dimensions (valence, arousal, and tiredness). The applied analytical procedure has been described in detail elsewhere (Lazarides et al., 2020). In four separate 4-level random intercept LMMs, the percentage of total variance for pregnancy-specific distress, valence, arousal, and tiredness was computed at the level of the momentary measurements (level 1), days (level 2), stages of pregnancy (level 3), and participants (level 4). To account for the unequal spacing of the auto-correlated measurements across a day a continuous time-autoregressive covariance structure of order one was specified using time since wake in minutes (Goldstein, Healy, & Rasbash, 1994; Jones, 1993; Littell, Milliken, Stroup, Wolfinger, & Oliver, 2006). Restricted maximum likelihood was used for parameter estimation (for R code, see online Supplement A1).

#### Linear mixed models

***EMA-based measures.*** To examine the effect of history of prenatal loss on pregnancy-specific distress and momentary maternal mood along the three dimensions valence, arousal, and tiredness, four separate 4-level LMMs were fitted to the nested data with the same random effect structure as described for the variance decomposition of EMA-based measures. We used the same continuous-autoregressive covariance structure to account for unequal temporal spacing of the momentary measurements. The exemplary R code is provided in online Supplement A2. Prenatal loss status (history of prenatal loss yes/no) was used as a dichotomous predictor. Relevant covariates (described below) were included as fixed effects in all models.

***Moderation by stage of pregnancy.*** To explore how potential differences in psychological state between women with and without a history of prenatal loss may change with advancing gestation, we included the interaction term between stage of pregnancy (i.e. visit number, *T*1 and *T*2) and prenatal loss status in the linear mixed models described above (R code, see online Supplement A3).

***Sensitivity analysis.*** The control group included women who were pregnant for the first time and women who were pregnant at least one time before the current pregnancy, whereas the group of women with a history of prenatal loss included only multigravida (multigravida = women that were pregnant at least once before). Because the experience of prior pregnancy may be associated with biological and psychological changes (Armstrong et al., 2009), this could introduce heterogeneity in the control group and limit the validity of reported differences in psychological well-being between women with and without prenatal loss. We therefore conducted a sensitivity analysis by testing our hypothesis in only multi-gravid women.

#### Covariates

All analyses were adjusted for the effects of potential confounders that have previously been associated with the risk for prenatal loss and impaired psychological well-being, including maternal age, parity (not included as a covariate in the sensitivity analyses), obstetric risk factors, and income (Magnus, Wilcox, Morken, Weinberg, & Haberg, 2019). The following covariates were included as fixed effects in all described LMMs (R code, see online Supplement A1): stage of pregnancy (early – T1 *v.* late pregnancy – T2), maternal age at first visit, income, parity category (0 – nulliparous, 1 – multiparous; not included in sensitivity analyses described above), obstetric risk factor (presence of any of the following conditions during the current pregnancy: preeclampsia, hypertension, gestational diabetes coded with ‘1’, no obstetric risk factors present coded with ‘0’).

#### Compliance and handling of missing data

Given the EMA protocol, each time a participant refrained from answering a prompt, declined to answer, ignored a prompt or did not conclude the entire survey, the smartphone application recorded a missing value. To assess compliance, we calculated the percent of missing prompts of the total number of prompts. In the statistical analyses, missing data were accounted for by use of full information restricted maximum likelihood estimation (Little & Rubin, 2002; Raudenbush & Bryk, 2002). Thus, LMMs make use of all available data.

## Results

### EMA-based measures of maternal psychological state

#### Compliance

Compliance with the EMA protocol (number of missing prompts relative to the total number of prompts) was 86.3%, which is above the recommended 80% for EMA-studies, and also above average compliance of 75–78% previously reported in two meta-analyses comprised of 168 EMA studies (Jones et al., 2019; Wen, Schneider, Stone, & Spruijt-Metz, 2017).

#### Variance decomposition

The variance decomposition indicates the amount of the total variation in pregnancy-specific distress, valence, arousal, and tiredness that is derived from the different levels of the data (i.e. variation between individuals as well as within individuals, across the stages of pregnancy, across a day, and across moments; de Haan-Rietdijk, Kuppens, and Hamaker, 2016; Schmiedek, Lovden, and Lindenberger, 2013). Based on the LMM, pregnancy-specific distress scores varied largely between individuals (68.4%) and to a lesser extent from moment to moment (12.1%), and from early to late pregnancy (16.9%), as well as to a small degree from day to day (5.1%; summary of results is given in online Supplementary Table S1, detailed results in online Supplementary Table S2). For the mood scales, GB_mean, CN_mean, and AT_mean, predominantly showed variation at the momentary level (48.8–54.3%) and between individuals (27.6–38.9%) rather than from day to day (7.1–10.2%) or from early to late pregnancy (3.7–5.9%; summary of results is given in online Supplementary Table S1, detailed results in online Supplementary Tables S3–S5). Intraclass correlation coefficients reflected this pattern of variation (online Supplementary Table S1).

#### Descriptive statistics

Summary statistics for pregnancy-specific distress and for each MDBF scale (valence, arousal, tiredness) are displayed separately for each time point (stage of pregnancy) in Table 2. Pregnancy-specific distress decreased slightly from early to late pregnancy in the whole sample. In general, mood improved from early to late gestation, as suggested by a decrease in mood valence (GB_mean), arousal (CN_mean) and level of tiredness (AT_mean) in the whole sample. The observed average and variation of mood scores are comparable with published norms for women in reproductive age (Hinz et al., 2012; Steyer et al., 1994; Steyer, Schwenkmezger, Notz, & Eid, 1997).

**Table 2.** Summary statistics on valence (good – bad mood: GB_mean), arousal (calm – nervous: CN_mean), tiredness (alert – tired: AT_mean), and pregnancy-specific distress (PSD_mean), separately for each time point, T1 and T2

	T1 (*N* = 155)		T2 (*N* = 104)	
Parameter	*M*	s.d.	*M*	s.d.
PSD_mean	1.24	0.891	1.19	0.917
GB_mean	1.41	0.957	1.22	0.920
CN_mean	1.67	0.947	1.49	0.941
AT_mean	2.11	1.04	2.01	1.16

*M,* Mean; s.d., Standard deviation; *N,* Sample size at measurement time point.

### History of prenatal loss and EMA measures of psychological state during pregnancy

An overview of the results of the linear mixed-effects model analysis is displayed in Table 3. More detailed results for each outcome are presented in online Supplementary Tables S6–S9.

**Table 3.** Results of linear mixed models predicting EMA-based pregnancy-specific distress (PSD_mean), and affective states (valence, GB_mean; arousal, CN_mean, tiredness, AT_mean) by stage of pregnancy and prenatal loss status

Fixed effects			
	*B* (s.e.)	95% CI for *B*	*p*
PSD_mean			
Intercept	1.803 (0.471)	0.879–2.727	<0.001***
Prenatal loss	0.465 (0.159)	0.151–0.779	0.004**
Stage of pregnancy	<−0.001 (0.054)	−0.106 to 0.106	0.997
GB_mean			
Intercept	1.264 (0.347)	0.584–1.944	<0.001***
Prenatal loss	0.213 (0.117)	−0.018 to 4.446	0.070 .
Stage of pregnancy	−0.110 (0.035)	−0.180 to −0.040	0.003**
CN_mean			
Intercept	1.661 (0.367)	0.943–2.380	<0.001***
Prenatal loss	0.247 (0.124)	0.003–0.491	0.047*
Stage of pregnancy	−0.118 (0.039)	−0.195 to −0.042	0.003**
AT_mean			
Intercept	2.323 (0.386)	1.566–3.080	<0.001***
Prenatal loss	0.293 (0.130)	0.036–0.550	0.026 *
Stage of pregnancy	−0.100 (0.049)	−0.197 to −0.004	0.042 *

*Note:* Significance codes: *p* > 0.01 ‘ ’, *p* < 0.10 ‘.’, *p* < 0.05 ‘*’, *p* < 0.01 ‘**’, *p* < 0.001 ‘***’. Results displayed for log-transformed cortisol. Transformation did not change magnitude, direction nor significance level of the reported effects. For fit indices see online Supplementary Table S10.

#### Pregnancy-specific distress

There was a significant effect of history of prenatal loss on pregnancy-specific distress: women with a history of prenatal loss reported significantly higher levels of pregnancy-specific distress assessed at a momentary level, in early as well as in late gestation (*B* = 0.465, *p* = 0.004, online Supplementary Table S6). On average, women with a history of prenatal loss reported 0.465-unit higher levels of pregnancy-specific distress on a scale from to those without a history of prenatal loss.

#### Mood valence, arousal, and tiredness

There was no significant main effect of prenatal loss status on mood valence (GB_mean) albeit mood was slightly impaired in women with a history of prenatal loss compared to those without across the course of pregnancy (*B* = 0.214, *p* = 0.070, online Supplementary Table S7). Arousal was positively associated with prenatal loss status (CN_mean: *B* = 0.247, *p* = 0.047, online Supplementary Table S8): across gestation, women with a history of prenatal loss showed increased arousal compared to women without prenatal loss, and were more tired (AT_mean: *B* = 0.293, *p* = 0.026, online Supplementary Table S9). Women with prenatal loss showed on average a 0.247-unit higher level of arousal and a 0.270-unit higher level of tiredness.

### Moderation of the association between history of prenatal loss and EMA measures of psychological state by stage of pregnancy

**Fig. 1.**
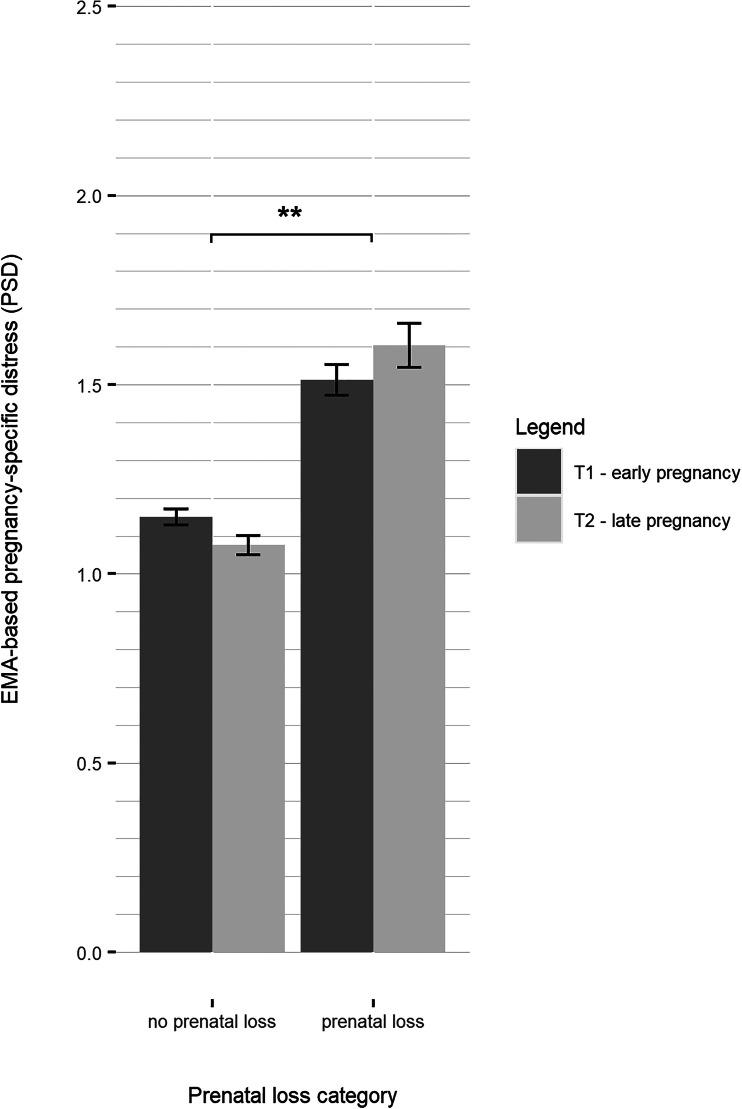
Grouped bar plot of EMA-based pregnancy-specific distress by history of prenatal loss in previous pregnancy and stage of pregnancy (mean ± 2 standard error bars).

There was no main effect of stage of pregnancy on levels of pregnancy-specific distress (PSD_mean: *B* = −0.0002, *p* = 0.997, online Supplementary Table S6). However, the moderation analysis revealed a significant positive interaction effect between stage of pregnancy and prenatal loss status (PSD_mean: *B* = 0.314, *p* = 0.012). As displayed in Fig. 1, pregnancy-specific distress decreased from early to late gestation in women without a history of prenatal loss, whereas it increased in women with a history of prenatal loss.

**Fig. 2.**
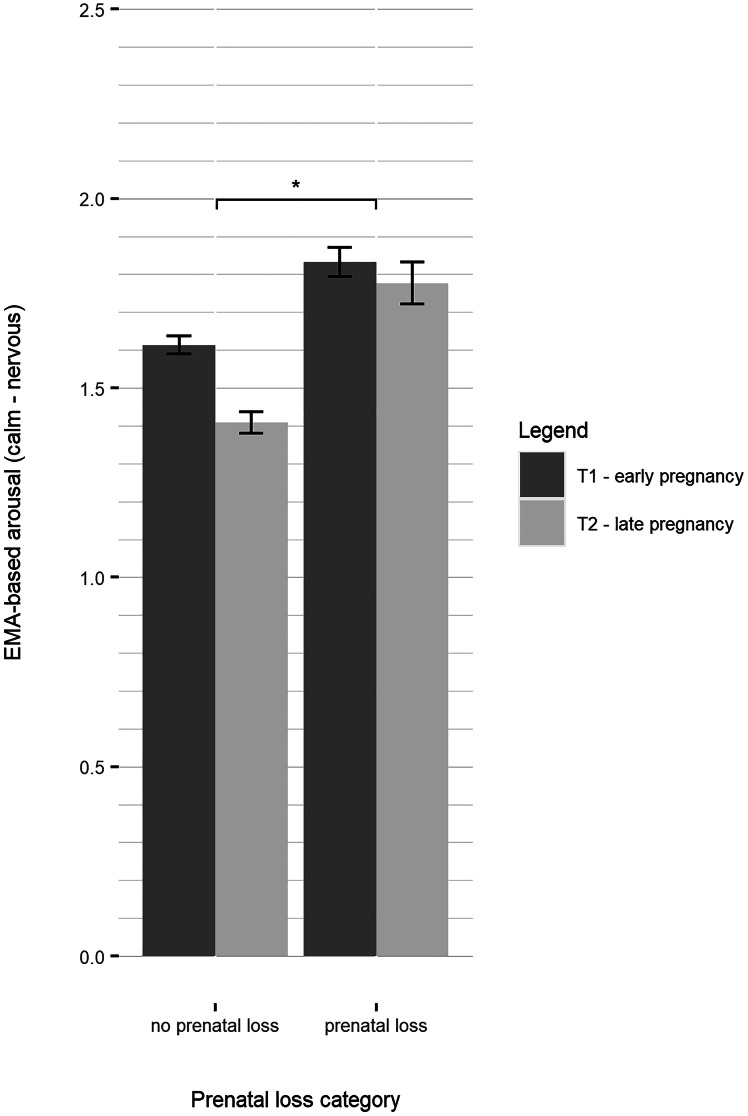
Grouped bar plot displaying EMA-based arousal by history of prenatal loss in previous pregnancy and stage of pregnancy (mean ± 2 standard error bars).

Across the whole sample, momentary maternal mood including valence (impaired mood), arousal, and tiredness decreased significantly across pregnancy (GB_mean: *B* = −0.110, *p* = 0.003; CN_mean: *B* = −0.118, *p* = 0.003; AT_mean: *B* = −0.100, *p* = 0.042; online Supplementary Tables S7–S9), indicating a general improvement of mood across the whole sample. We therefore conducted a moderation analysis to test if the effect of prenatal loss on mood dimensions was moderated by the stage of pregnancy. The moderation analysis revealed a significant interaction effect of stage of pregnancy and prenatal loss status on arousal (CN_mean: *B* = 0.184, *p* = 0.045, Fig. 2). Women without a history of prenatal loss reported lower levels of arousal in late compared to early gestation, while levels did not decrease in women with a history of prenatal loss. There was no significant interaction effect of stage of pregnancy and prenatal loss status on mood valence and tiredness (GB_mean: *B* = 0.142, *p* = 0.090; AT_mean: *B* = 0.116, *p* = 0.317).

### Sensitivity analysis

When considering only women who were pregnant at least one time before the current pregnancy (40 women with and 39 without a history of prenatal loss), most of the previously reported effects remained unchanged in direction, magnitude, and significance level. Specifically, women with a history of prenatal loss reported higher levels of pregnancy-specific distress (PSD_mean: *B* = 0.482, *p* = 0.039), and were more nervous (CN_mean: *B* = 0.359, *p* = 0.037), more tired (AT_mean: *B* = 0.494, *p* = 0.003), across pregnancy compared to women without prenatal loss. Furthermore, there was a trend for an effect of prenatal loss status on mood valence when only including multigravid women. Women with a history of prenatal loss reported impaired mood compared to women without prenatal loss (GB_mean: *B* = 0.294, *p* = 0.081).

## Discussion

To the best of our knowledge, this is the first study to use the EMA approach to comprehensively (i.e. serially, at a momentary level, across everyday life situations) quantify and compare stress and mood levels and trajectories in pregnancy in women with and without a prior history of prenatal loss. Our findings indicate that women with a prior history of prenatal loss experienced significantly more pregnancy-related stress and felt significantly more nervous and tired compared to those who have not previously experienced a prenatal loss. Moreover, our results suggest that these differences persisted and even grew or became amplified as pregnancy progressed. Maternal levels of stress and negative affect progressively decreased over the course of pregnancy in women without a history of prenatal loss, whereas they did not change or even increased in women with a prior history of prenatal loss. The magnitude of this observed difference is striking. Women in the prior prenatal loss group exhibited, on average, 38.6% more pregnancy-specific stress, 18.3% more arousal, and 15.5% more exhaustion than those in the comparison group^†^^1^. The present study was not designed to address the clinical relevance of observed findings. Because the vast majority of studies of the effects of maternal stress in pregnancy have relied on the more traditional retrospective recall approach to quantify stress, as opposed to the EMA approach used in the current study, it is difficult to directly extrapolate clinical significance. We note, nevertheless, that several previous studies have reported that differences of comparable or even smaller magnitude in maternal stress during and/or across pregnancy have been independently associated with a range of adverse maternal, birth and child developmental and health outcomes, including premature birth, newborn and infant adiposity, neurodevelopmental deficits, and even cellular measures of aging (telomere length) (Buss et al., 2012b; Entringer et al., 2018; Gyllenhammer, Entringer, Buss, & Wadhwa, 2020; Lindsay, Buss, Wadhwa, & Entringer, 2018; Wadhwa et al., 2011). Based on this observation we submit it is likely that the magnitude of the observed difference in maternal stress in the current study may portend clinical significance.

Our results are consistent with those of previous studies that find women report increased levels of post-traumatic stress, anxiety, and depression following pregnancy loss (Farren et al., 2016, 2020; Hughes et al., 1999; Turton et al., 2009). Across time, grief subsides and psychiatric disorders possibly remit, although, the emotional perturbations related to the experience of pregnancy loss remain (Kersting et al., 2007; Krosch & Shakespeare-Finch, 2017; Volgsten et al., 2018). A subsequent pregnancy has the potential to reactivate the affective memories associated with the past prenatal loss (Haas & Canli, 2008). The current study highlights the relevance of history of prenatal loss as a risk factor for increased prenatal stress in pregnancy and thus the potential negative consequences on pregnancy, birth and child development, and health outcomes.

In the current study, we observed an overall improvement across pregnancy in psychological well-being. This observation is consistent with recent evidence from a clinical population of pregnant women, who reported a decrease in psychopathological symptoms from early to late pregnancy (Asselmann, Kunas, Wittchen, & Martini, 2020). This general improvement of well-being and decrease in psychological stress may be associated with the attenuation of maternal biological stress responsivity across pregnancy (Entringer et al., 2010). However, in our study, women with a history of prenatal loss did not exhibit this decrease in stress and improvement in mood across pregnancy, which may be a consequence of their prior traumatic experience of losing a pregnancy and the resultant general feeling of uncontrollability. We have previously reported that the lack of stress attenuation across pregnancy is related to adverse pregnancy outcomes (Buss et al., 2009).

We suggest that our study has several strengths. As of our knowledge, this is the first study to assess the effect of a history of prenatal loss on maternal psychological state in early and late pregnancy. We use EMA methods to assess maternal stress in women's everyday life in natural settings in contrast to previous research that exclusively relied on traditional retrospective questionnaires. Participants of the current study showed a high compliance with the EMA protocol. We assessed psychological state on a continuum, and relied on measures of mood and stress rather than focusing on clinical symptom categories or psychiatric diagnoses. We adjusted our analyses for the effect of important potential confounders, including sociodemographic factors, obstetric characteristics and number of previous pregnancies. By means of a sensitivity analysis within multigravida women only, we confirm the robustness of our results.

Some limitations of the current study need to be acknowledged. First, data on psychological state was available only for the current pregnancy, measures of psychological state prior to the initial prenatal loss were not available. Stress has been discussed as a risk factor for prenatal loss. However, previous studies investigating whether maternal psychological stress predicts prenatal loss have produced mixed results (Klebanoff, Shiono, & Rhoads, 1990; Milad, Klock, Moses, & Chatterton, 1998; Nelson et al., 2003; Qu et al., 2017). All our analyses were adjusted for covariates potentially associated with both risk for prenatal loss and maternal psychological state, including maternal age, parity, obstetric risk, and household income. Second, we were unable to test the effect of the number of previous prenatal losses on psychological state during pregnancy due to the relatively small number of women who miscarried more than once. Previous large cohort studies suggest that with increasing number of prenatal losses women report even higher levels of depression and anxiety (Blackmore et al., 2011; Toffol, Koponen, & Partonen, 2013). We therefore submit that our findings may represent a conservative estimate of the true effect of history of prenatal loss on psychological state during pregnancy. Third, data on the length of inter-pregnancy intervals as well as on a whether or not women gave birth to a living child between the pregnancy loss and the current pregnancy were not available, and we were therefore unable to test the moderating effects of these variables. Previous studies suggest that the length of the inter-pregnancy interval does not affect the association between history of prenatal loss and depression and/or anxiety during a subsequent pregnancy or in the postpartum period (Gravensteen et al., 2018; Schetter, Saxbe, Cheadle, & Guardino, 2016). A large longitudinal cohort study reports a robust association between history of prenatal loss with increased levels of anxiety and depression during a subsequent pregnancy, which remained stable across the pre- and postnatal period of the index pregnancy, thereby indicating that the psychological impairment associated with previous prenatal loss might not attenuate significantly following the birth of a living child (Blackmore et al., 2011). Fourth, EMA studies assess the participants' psychological state repeatedly across multiple days raising the issue of measurement reactivity. However, previous EMA studies have provided no evidence for measurement reactivity with regard to mood, craving, body image, and suicidal thoughts (Coppersmith, 2020; De Vuyst, Dejonckheere, Van der Gucht, & Kuppens, 2019; Heron & Smyth, 2013; Hufford, Shields, Shiffman, Paty, & Balabanis, 2002; Rowan et al., 2007).

The findings of our study suggest that women with a history of prenatal loss are at increased risk for experiencing higher levels of stress during pregnancy. Although, obstetric guidelines issued by the American College of Obstetricians and Gynecologists advice perinatal care providers to screen for postpartum depression, recommendations to not include screening for psychological stress (American Academy of Pediatrics & American College of Obstetricians and Gynecologists, 2017). Our results imply that pregnancy-specific distress might be a good screening tool for this purpose, since the effect on pregnancy loss on pregnancy specific distress in our study was substantial, and it primarily varied between individuals and may not have to be measured that frequently.

The current study underscores the importance of using EMA methods in assessing stress and mood in the context of pregnancy, which then could be used to design personalized interventions to reduce maternal stress. EMA-based measures of psychological states can be used to estimate subject-specific ‘moments at risk’, such as the deviation from the individual average stress level in a given moment, that have a higher predictive value for maternal cortisol levels during pregnancy than traditional approaches (Lazarides et al., 2020). Future studies could test the efficacy of EMA-based targeted interventions in women with a history of prenatal loss. Given its substantial burden on maternal and offspring health, reducing stress during pregnancy in this high risk group could yield considerable public health benefit.

## Footnotes

† The notes appear after the main text.

## Footnotes

1 The reported percentages are based on the group means of the respective outcomes across measurement time points. These effect quantifications do not account for the nesting of the data, group sizes, variation within groups, and potential effect of covariates which were rather small in this sample. Therefore, these approximate effect sizes should be interpreted under these limitations.

## Supplementary material

For supplementary material accompanying this paper visit https://doi.org/10.1017/S0033291721002221. click here to view supplementary material
