# Supplementary material for: The association between history of prenatal loss and maternal psychological state in a subsequent pregnancy: an ecological momentary assessment (EMA) study
Source: Psychol Med. 2021 Jun 15;53(3):855–65. doi: 10.1017/S0033291721002221 (PMC9975992; doi:10.1017/S0033291721002221)
Supplement: Supplementary file 1 [file S0033291721002221sup001.docx]

# Supplement

**Supplement A1: R code variance decomposition**

PSD_mean $=$ Average EMA-based pregnancy specific distress score in a given moment *m*, on a given day *d*, in a stage of pregnancy *t*, in an individual *i*

library(nlme)

library(mlmRev)

ri00a <- lme(fixed = PSD_mean ~ 1,

method = "REML",

random = list(ID=~1, MeasurementTime=~1, Day=~1),

data = data,

control = lmeControl(opt = "optim"),

correlation = corCAR1(form= ~ 1|ID/Stage of pregnancy/Day/time_since_wake_min),

na.action = na.omit)

GB_mean_rev $=$ Average EMA-based good-bad mood (valence) score in a given moment *m*, on a given day *d*, in a stage of pregnancy *t*, in an individual *i*

library(nlme)

library(mlmRev)

ri00a <- lme(fixed = GB_mean_rev ~ 1,

method = "REML",

random = list(ID=~1, MeasurementTime=~1, Day=~1),

data = data,

control = lmeControl(opt = "optim"),

correlation = corCAR1(form= ~ 1|ID/Stage of pregnancy/Day/time_since_wake_min),

na.action = na.omit)

CN_mean_rev $=$ Average EMA-based calm-nervous mood (arousal/nervousness) score in a given moment *m*, on a given day *d*, in a stage of pregnancy *t*, in an individual *i*

library(nlme)

library(mlmRev)

ri00a <- lme(fixed = CN_mean_rev ~ 1,

method = "REML",

random = list(ID=~1, MeasurementTime=~1, Day=~1),

data = data,

control = lmeControl(opt = "optim"),

correlation = corCAR1(form= ~ 1|ID/Stage of pregnancy/Day/time_since_wake_min),

na.action = na.omit)

AT_mean_rev $=$ Average EMA-based awake-tired mood (tiredness) score in a given moment *m*, on a given day *d*, in a stage of pregnancy *t*, in an individual *i*

library(nlme)

library(mlmRev)

ri00a <- lme(fixed = AT_mean_rev ~ 1,

method = "REML",

random = list(ID=~1, MeasurementTime=~1, Day=~1),

data = data,

control = lmeControl(opt = "optim"),

correlation = corCAR1(form= ~ 1|ID/Stage of pregnancy/Day/time_since_wake_min),

na.action = na.omit)

**Supplement A2: R Code fixed effect of prenatal loss status and stage of pregnancy on EMA-based assessments**

PSD_mean $=$ Average EMA-based pregnancy-specific distress score in a given moment *m*, on a given day *d*, in a stage of pregnancy *t*, in an individual *i*

library(nlme)

library(mlmRev)

ri00a <- lme(fixed = PSD_mean ~ prenatal loss_status + MeasurementTime +

age_atfirstvisit + i_parity_cat + f_income + obrisk_cat,

method = "REML",

random = list(ID=~1, MeasurementTime=~1, Day=~1),

data = data,

control = lmeControl(opt = "optim"),

correlation = corCAR1(form= ~ 1|ID/Stage of pregnancy/Day/time_since_wake_min),

na.action = na.omit)

GB_mean_rev $=$ Average EMA-based good-bad mood (valence) score in a given moment *m*, on a given day *d*, in a stage of pregnancy *t*, in an individual *i*

library(nlme)

library(mlmRev)

ri00a <- lme(fixed = GB_mean_rev ~ prenatal loss_status + MeasurementTime +

age_atfirstvisit + i_parity_cat + f_income + obrisk_cat,

method = "REML",

random = list(ID=~1, MeasurementTime=~1, Day=~1),

data = data,

control = lmeControl(opt = "optim"),

correlation = corCAR1(form= ~ 1|ID/Stage of pregnancy/Day/time_since_wake_min),

na.action = na.omit)

CN_mean_rev $=$ Average EMA-based calm-nervous mood (arousal/nervousness) score in a given moment *m*, on a given day *d*, in a stage of pregnancy *t*, in an individual *i*

library(nlme)

library(mlmRev)

ri00a <- lme(fixed = CN_mean_rev ~ prenatal loss_status + MeasurementTime +

age_atfirstvisit + i_parity_cat + f_income + obrisk_cat,

method = "REML",

random = list(ID=~1, MeasurementTime=~1, Day=~1),

data = data,

control = lmeControl(opt = "optim"),

correlation = corCAR1(form= ~ 1|ID/Stage of pregnancy/Day/time_since_wake_min),

na.action = na.omit)

AT_mean_rev $=$ Average EMA-based awake-tired mood (tiredness) score in a given moment *m*, on a given day *d*, in a stage of pregnancy *t*, in an individual *i*

library(nlme)

library(mlmRev)

ri00a <- lme(fixed = AT_mean_rev ~ prenatal loss_status + MeasurementTime +

age_atfirstvisit + i_parity_cat + f_income + obrisk_cat,

method = "REML",

random = list(ID=~1, MeasurementTime=~1, Day=~1),

data = data,

control = lmeControl(opt = "optim"),

correlation = corCAR1(form= ~ 1|ID/Stage of pregnancy/Day/time_since_wake_min),

na.action = na.omit)

**Supplement A3: R code interaction effect of prenatal loss status and stage of pregnancy on EMA-based assessments**

PSD_mean $=$ Average EMA-based pregnancy-specific distress score in a given moment *m*, on a given day *d*, in a stage of pregnancy *t*, in an individual *i*

library(nlme)

library(mlmRev)

ri00a <- lme(fixed = PSD_mean ~ prenatal loss_status* MeasurementTime +

age_atfirstvisit + i_parity_cat + f_income + obrisk_cat,

method = "REML",

random = list(ID=~1, MeasurementTime=~1, Day=~1),

data = data,

control = lmeControl(opt = "optim"),

correlation = corCAR1(form= ~ 1|ID/Stage of pregnancy/Day/time_since_wake_min),

na.action = na.omit)

GB_mean_rev $=$ Average EMA-based good-bad mood (valence) score in a given moment *m*, on a given day *d*, in a stage of pregnancy *t*, in an individual *i*

library(nlme)

library(mlmRev)

ri00a <- lme(fixed = GB_mean_rev ~ prenatal loss_status*MeasurementTime +

age_atfirstvisit + i_parity_cat + f_income + obrisk_cat,

method = "REML",

random = list(ID=~1, MeasurementTime=~1, Day=~1),

data = data,

control = lmeControl(opt = "optim"),

correlation = corCAR1(form= ~ 1|ID/Stage of pregnancy/Day/time_since_wake_min),

na.action = na.omit)

CN_mean_rev $=$ Average EMA-based calm-nervous mood (arousal/nervousness) score in a given moment *m*, on a given day *d*, in a stage of pregnancy *t*, in an individual *i*

library(nlme)

library(mlmRev)

ri00a <- lme(fixed = CN_mean_rev ~ prenatal loss_status*MeasurementTime +

age_atfirstvisit + i_parity_cat + f_income + obrisk_cat,

method = "REML",

random = list(ID=~1, MeasurementTime=~1, Day=~1),

data = data,

control = lmeControl(opt = "optim"),

correlation = corCAR1(form= ~ 1|ID/Stage of pregnancy/Day/time_since_wake_min),

na.action = na.omit)

AT_mean_rev $=$ Average EMA-based awake-tired mood (tiredness) score in a given moment *m*, on a given day *d*, in a stage of pregnancy *t*, in an individual *i*

library(nlme)

library(mlmRev)

ri00a <- lme(fixed = AT_mean_rev ~ prenatal loss_status*MeasurementTime +

age_atfirstvisit + i_parity_cat + f_income + obrisk_cat,

method = "REML",

random = list(ID=~1, MeasurementTime=~1, Day=~1),

data = data,

control = lmeControl(opt = "optim"),

correlation = corCAR1(form= ~ 1|ID/Stage of pregnancy/Day/time_since_wake_min),

na.action = na.omit)

Table S1.

Summary of decomposition of variance and intraclass correlation coefficients for EMA-based momentary measures.

|  | Parameter | | | | | | | |
| --- | --- | --- | --- | --- | --- | --- | --- | --- |
|  | **PSD_mean** | | **GB_mean** | | **CN_mean** | | **AT_mean** | |
| **ICC** | .771 | | .386 | | . 429 | | .327 | |
|  | Variance | Variance | % of total variance | Variance | Variance | % of total variance | Variance |  |
| Total | 0.813 | 0.898 |  | 0.900 | 0.898 |  | 0.900 |  |
| ID | 0.556 | 0.328 | 36.5 | 0.350 | 0.328 | 36.5 | 0.350 |  |
| Stage of Pregnancy | 0.137 | 0.033 | 3.7 | 0.051 | 0.033 | 3.7 | 0.051 |  |
| Day | 0.042 | 0.065 | 7.2 | 0.064 | 0.065 | 7.2 | 0.064 |  |
| Moment + Measurement Error | 0.099 | 0.488 | 54.3 | 0.440 | 0.488 | 54.3 | 0.440 |  |
| *Note.* Due to rounding, some totals may not correspond with the sum of the separate figures. No. = number. ICC = intraclass correlation coefficient. | | | | | | | | |

Table S2.

Variance Decomposition: **PSD_mean** (range: 0 – 5, ICC = .771)

| Level | No. of observations in group | Variance | % variance of total variance | |
| --- | --- | --- | --- | --- |
| Total | 6609 | 0.813 |  | |
| ID | 154 | 0.556 | 68.4 | |
| Measurement Time | 258 | 0.137 | 16.9 | |
| Day | 982 | 0.042 | 5.1 | |
| Moment + Measurement Error | 6609 | 0.099 | 12.1 | |
| *Note.* Due to rounding, some totals may not correspond with the sum of the separate figures. No. = number. | | | |  |

Table S3.

Variance Decomposition: GB_mean - **Valence** (range: 0 – 5, ICC = .386)

| Level | No. of observations in group | Variance | % variance of total variance | |
| --- | --- | --- | --- | --- |
| Total | 6585 | 0.898 |  | |
| ID | 154 | 0.328 | 36.5 | |
| Measurement Time | 258 | 0.033 | 3.7 | |
| Day | 982 | 0.065 | 7.2 | |
| Moment + Measurement Error | 6585 | 0.488 | 54.3 | |
| *Note.* Due to rounding, some totals may not correspond with the sum of the separate figures. No. = number. | | | |  |

Table S4.

Variance Decomposition: CN_mean - **Arousal** (range: 0 – 5, ICC = .429)

| Level | No. of observations in group | Variance | % variance of total variance | |
| --- | --- | --- | --- | --- |
| Total | 6578 | 0.900 |  | |
| ID | 154 | 0.350 | 38.9 | |
| Measurement Time | 258 | 0.051 | 5.6 | |
| Day | 982 | 0.064 | 7.1 | |
| Moment + Measurement Error | 6578 | 0.440 | 48.8 | |
| *Note.* Due to rounding, some totals may not correspond with the sum of the separate figures. No. = number. | | | |  |

Table S5.

Variance Decomposition: AT_mean - **Tiredness** (range: 0 – 5; ICC = .327)

| Level | No. of observations in group | Variance | % variance of total variance | |
| --- | --- | --- | --- | --- |
| Total | 6581 | 1.189 |  | |
| ID | 154 | 0.328 | 27.6 | |
| Measurement Time | 258 | 0.071 | 5.9 | |
| Day | 982 | 0.121 | 10.2 | |
| Moment + Measurement Error | 6581 | 0.646 | 54.4 | |
| *Note.* Due to rounding, some totals may not correspond with the sum of the separate figu4res. No. = number. | | | |  |

Table S6.

Results of LMM for pregnancy-specific distress (PSD_mean) predicted by prenatal loss status, stage of pregnancy, parity category, income, obstetric risk factor category, and maternal age at first visit as fixed effects, random effects structure of momentary measurements within days in stage of pregnancy nested in participants.

| **Fixed effects** | | | | |
| --- | --- | --- | --- | --- |
|  | *B* (*SE*) |  | 95% CI for *B* | *p* |
| Intercept | 1.803 (0.471) |  | 0.879 – 2.727 | <.001*** |
| Level 4 |  |  |  |  |
| Prenatal loss status | 0.465 (0.159) |  | 0.151 – 0.779 | .004** |
| Parity | 0.012 (0.144) |  | -0.272 – 0.295 | .936 |
| Income | <0.001 (<0.001) |  | [<-0.001 - <0.001] | .132 |
| Obstetric risk | 0.012 (0.267) |  | -0.515 – 0.539 | .965 |
| Maternal age | -0.015 (0.016) |  | -0.046 – 0.017 | .358 |
| Level 3 |  |  |  |  |
| Stage of pregnancy | <-0.001 (0.054) |  | -0.106 – 0.106 | .997 |
| Level 2 |  |  |  |  |
| Level 1 |  |  |  |  |
| **Random effects** | | | | |
|  | Variance |  | *SD* |  |
| ID | 0.505 |  | 0.711 |  |
| Stage of pregnancy | 0.138 |  | 0.372 |  |
| Day | 0.042 |  | 0.205 |  |
| Residual | 0.097 |  | 0.311 |  |
| *Note.* Significance codes: *p* > .01 ‘ ’ *p* < .10 ‘.’ *p* < .05 ‘*’*p* < 0.01 ‘**’ *p* < .001 ‘***’. For fit indices: AIC = 5390.089, BIC = 5471.249, logLik = -2683.045. | | | | |

Table S7.

Results of LMM for momentary mood – valence (good - bad) predicted by prenatal loss status, stage of pregnancy, parity category, income, obstetric risk factor category, and maternal age at first visit as fixed effects, random effects structure of momentary measurements within days in stage of pregnancy nested in participants.

| **Fixed effects** | | | | |
| --- | --- | --- | --- | --- |
|  | *B* (*SE*) |  | 95% CI for *B* | *p* |
| Intercept | 1.264 (0.347) |  | 0.584 – 1.944 | <.001*** |
| Level 4 |  |  |  |  |
| Prenatal loss status | 0.213 (0.117) |  | -0.018 – 0.445 | .070 . |
| Parity | 0.086 (0.105) |  | -0.122 – 0.296 | .413 |
| Income | <-0.001 (<0.001) |  | [<-0.001 - <0.001] | .055 . |
| Obstetric risk | -0.066 (0.196) |  | -0.453 – 0.321 | .737 |
| Maternal age | 0.008 (0.012) |  | -0.015 – 0.031 | .508 |
| Level 3 |  |  |  |  |
| Stage of pregnancy | -0.110 (0.035) |  | -0.180 – -0.040 | .003** |
| Level 2 |  |  |  |  |
| Level 1 |  |  |  |  |
| **Random effects** | | | | |
|  | Variance |  | *SD* |  |
| ID | 0.284 |  | 0.533 |  |
| Stage of pregnancy | 0.028 |  | 0.168 |  |
| Day | 0.066 |  | 0.257 |  |
| Residual | 0.489 |  | 0.699 |  |
| *Note.* Significance codes: *p* > .01 ‘ ’ *p* < .10 ‘.’ *p* < .05 ‘*’*p* < 0.01 ‘**’ *p* < .001 ‘***’. For fit indices: AIC = 14640.02, BIC = 14721.14, logLik = -7308.012. | | | | |

Table S8.

Results of LMM for momentary mood – arousal (calm – nervous) predicted by prenatal loss status, stage of pregnancy, parity category, income, obstetric risk factor category, and maternal age at first visit as fixed effects, random effects structure of momentary measurements within days in stage of pregnancy nested in participants.

| **Fixed effects** | | | | |
| --- | --- | --- | --- | --- |
|  | *B* (*SE*) |  | 95% CI for *B* | *p* |
| Intercept | 1.661 (0.367) |  | 0.943 – 2.380 | <.001*** |
| Level 4 |  |  |  |  |
| Prenatal loss status | 0.247 (0.124) |  | 0.003 – 0.491 | .047* |
| Parity | 0.057 (0.112) |  | -0.163 – 0.278 | .608 |
| Income | <-0.001 (<0.001) |  | [<-0.001 – <0.001] | .355 |
| Obstetric risk | 0.041 (0.207) |  | -0.368 – 0.450 | .737 |
| Maternal age | 0.001 (0.012) |  | -0.023 – 0.026 | .925 |
| Level 3 |  |  |  |  |
| Stage of pregnancy | -0.118 (0.039) |  | -0.195 – -0.049 | .003** |
| Level 2 |  |  |  |  |
| Level 1 |  |  |  |  |
| **Random effects** | | | | |
|  | Variance |  | *SD* |  |
| ID | 0.313 |  | 0.569 |  |
| Stage of pregnancy | 0.044 |  | 0.209 |  |
| Day | 0.065 |  | 0.255 |  |
| Residual | 0.439 |  | 0.662 |  |
| *Note.* Significance codes: *p* > .01 ‘ ’ *p* < .10 ‘.’ *p* < .05 ‘*’*p* < 0.01 ‘**’ *p* < .001 ‘***’. For fit indices: AIC = 14030.69, BIC = 14111.79, logLik = -7003.343. | | | | |

Table S9.

Results of LMM for momentary mood – tiredness (alert – tired) predicted by prenatal loss status, stage of pregnancy, parity category, income, obstetric risk factor category, and maternal age at first visit as fixed effects, random effects structure of momentary measurements within days in stage of pregnancy nested in participants.

| **Fixed effects** | | | | |
| --- | --- | --- | --- | --- |
|  | *B* (*SE*) |  | 95% CI for *B* | *p* |
| Intercept | 2.323 (0.386) |  | 1.566 – 3.080 | <.001*** |
| Level 4 |  |  |  |  |
| Prenatal loss status | 0.293 (0.130) |  | 0.036 – 0.550 | .026 * |
| Parity | 0.182 (0.117) |  | -0.050 – 0.414 | .123 |
| Income | <0.001 (<0.001) |  | [<-0.001 – <0.001] | .861 |
| Obstetric risk | -0.030 (0.217) |  | -0.400 - 0.050 | .892 |
| Maternal age | -0.011 (0.013) |  | -0.036 – 0.015 | .415 |
| Level 3 |  |  |  |  |
| Stage of pregnancy | -0.100 (0.049) |  | 0.042 – 0.195 | .042 * |
| Level 2 |  |  |  |  |
| Level 1 |  |  |  |  |
| **Random effects** | | | | |
|  | Variance |  | *SD* |  |
| ID | 0.322 |  | 0.568 |  |
| Stage of pregnancy | 0.069 |  | 0.263 |  |
| Day | 0.117 |  | 0.342 |  |
| Residual | 0.644 |  | 0.802 |  |
| *Note.* Significance codes: *p* > .01 ‘ ’ *p* < .10 ‘.’ *p* < .05 ‘*’*p* < 0.01 ‘**’ *p* < .001 ‘***’. For fit indices: AIC = 16457.69, BIC = 16545.49, logLik = -8215.844. | | | | |
